# Sexually transmitted infections among artisanal miners in Zimbabwe: An urgent need for enhanced preventive measures

**DOI:** 10.1016/j.puhip.2022.100284

**Published:** 2022-06-18

**Authors:** Grant Murewanhema, Godfrey Musuka, Phanuel Tawanda Gwinji, Mathias Dzobo, Tafadzwa Dzinamarira

**Affiliations:** College of Medicine and Health Sciences, University of Zimbabwe, Harare, Zimbabwe; ICAP at Columbia University, Harare, Zimbabwe; College of Medicine and Health Sciences, University of Zimbabwe, Harare, Zimbabwe; School of Health Systems and Public Health, University of Pretoria, South Africa; ICAP at Columbia University, Harare, Zimbabwe; ICAP at Columbia University, Kigali, Rwanda

Dear Editor

Globally, more than one million sexually transmitted infections (STIs) are acquired and spread every day, most of which are asymptomatic [1]. *Neisseria gonorrhoea*, *Chlamydia trachomatis*, *Trichomonas vaginalis* and *Treponema pallidum* were the commonest sexually transmitted pathogens in Zimbabwe for many years, before the HIV epidemic. The majority of STIs are underdiagnosed and asymptomatic cases are often missed due to the reliance by most countries including Zimbabwe on syndromic management of the STIs [2]. The pathogens are of serious public health significance, causing major challenges at the individual and societal levels. At the individual level, short term problems include acute pelvic inflammatory disease (PID), which can be complicated with tubo-ovarian abscesses, peritonitis, septicaemia and even death, whereas the most significant long-term problems include pelvic adhesions, subfertility, ectopic pregnancies, and chronic pelvic pain. *T. pallidum* causes syphilis, which also has several complications including neurosyphilis and congenital syphilis in the newborn if untreated. The other main challenge associated with these STIs, both ulcerative and non-ulcerative, is the significantly increased risk of HIV acquisition among sufferers, particularly with herpes, gonorrhoea and syphilis [1]. Like many public health problems, the STI/HIV nexus is an important public health problem due to the complexity and multi-layered causal factors at various levels. Hence, measures to reduce the incidence of STIs have been critical for HIV control.

Over the past decade in Zimbabwe, significant strides have been made in reducing the incidence of both STIs and HIV. However, recent reports indicate that there is an increased incidence of these STIs in some informal mining areas of Zimbabwe [3]. Mining areas in the country, both informal and formal, have been traditionally perceived as hotspots for HIV and STI transmission. The actual magnitude of the problem is currently unknown; however, whatever level of transmission raises a significant concern as it serves to reverse the gains made over the past decade to reduce the burden of both STIs and HIV. Some of the drivers of cases of STIs among illegal miners as reported were poor sexual health knowledge/resistance to condom use, substance misuse, prolonged stay away from family, stigma, lack of entertainment, cultural status, and poor sexual health services [4]. An urgent evaluation of the extent of the problem and contributory factors is warranted; there is an urgent need for public health interventions to curb the scourge of STIs among artisanal miners in Zimbabwe.

There is a need for enhanced messaging on the danger of STIs on the different forms of media available in these areas including radio, television and social media platforms which are accessible to the informal miners and other people involved as they are relatively young. Additionally, attractive and compelling information education and communication (IEC) materials must be distributed widely in these areas at all strategic points where the people gather with strong reminders of the dangers of unprotected sexual intercourse, STIs, and HIV. Preventive services must be made readily available in these areas, including the distribution of male and female condoms for free, with support from developmental partners working in these areas. Additionally, it is important to set up clinics in this area where services for pre-exposure and post-exposure prophylaxis for HIV, screening, diagnosis and treatment for STIs, cervical cancer screening, and family planning services can be offered as an integrated package.

We furthermore recommend the application of Systems Thinking and creative problem-solving methodologies such as Soft Systems Methodology (SSM) which attempt to ensure that problems solved in the short term will remain in a solved state in the long term [5]. Systems thinking expands the range of choices available for problem-solving by encouraging holism, helping in the articulation of problems in new ways, giving a different worldview to the problem, compared to traditional reductionist approaches, and also includes active participation of all relevant stakeholders [5]. It is useful in the solving of complex problems that have a familiar and known history, are continuously recurring, and have been unsuccessfully solved using other methods before. Such problems as the upsurge of STIs in mining areas and other hotspots that are not amenable to the usual methods of problem-solving are worrisome and could push the health systems and other support functions to breaking point. They therefore require a paradigm shift that entails both thinking outside the box and making use of different modalities and methodologies.

Such control strategies should include contact tracing and expedited partner treatment for clients to break the chains of transmission. Widespread rollout of the different HIV pre-exposure prophylaxis regimens should be considered to cater for clients with diverse needs. Multipurpose prevention technologies that prevent HIV, STIs, and pregnancy would be the most ideal for these populations. Rapid and effective treatment of STIs is an important control strategy for HIV acquisition, and as these populations are high-risk for human papilloma virus transmission, contacts should not be missed opportunities to offer cervical cancer screening services to eligible women. Strategies to reduce stigma, a significant barrier to seeking healthcare are necessary to facilitate service utilisation. Of importance is education on the correct and consistent use of condoms and to demystify beliefs around condoms.

In conclusion, the control of STI transmission in artisanal mining areas is an urgent public health priority requiring an urgent intersection between all relevant stakeholders including the concerned communities to find effective and sustainable strategies. Additionally, primary research to quantify the magnitude of the problem and monitoring and evaluation frameworks to guide the control programs are needed. This allows measurement of the interventions’ success and provides strategic information to guide future programming.
